# Targeted Delivery of Cisplatin by Gold Nanoparticles: The Influence of Nanocarrier Surface Modification Type on the Efficiency of Drug Binding Examined by CE-ICP-MS/MS

**DOI:** 10.3390/ijms23042324

**Published:** 2022-02-19

**Authors:** Anna M. Wróblewska, Aleksandra Milewska, Marcin Drozd, Magdalena Matczuk

**Affiliations:** 1 Chair of Analytical Chemistry, Faculty of Chemistry, Warsaw University of Technology, Noakowskiego St. 3, 00-664 Warsaw, Poland; awroblewska@ch.pw.edu.pl (A.M.W.); aleksandra.milewska2.stud@pw.edu.pl (A.M.); 2 Chair of Medical Biotechnology, Faculty of Chemistry, Warsaw University of Technology, Noakowskiego St. 3, 00-664 Warsaw, Poland; mdrozd@ch.pw.edu.pl; 3 Center for Advanced Materials and Technologies, Warsaw University of Technology, Poleczki St. 19, 02-822 Warsaw, Poland

**Keywords:** cisplatin, gold nanoparticles, drug delivery, targeted drug delivery systems, nanoparticle surface modification, nanocarrier surface functionalization, capillary electrophoresis, mass spectrometry, hyphenated techniques, CE-ICP-MS

## Abstract

Spherical gold nanoparticles (GNPs), whose unique properties regarding biomedical applications were broadly investigated, are an object of interest as nanocarriers in drug targeted delivery systems (DTDSs). The possibility of surface functionalization, especially in enabling longer half-life in the bloodstream and enhancing cellular uptake, provides an opportunity to overcome the limitations of popular anticancer drugs (such as cisplatin) that cause severe side effects due to their nonselective transportation. Herein, we present investigations of gold nanoparticle–cisplatin systems formation (regarding reaction kinetics and equilibrium) in which it was proved that the formation efficiency and stability strongly depend on the nanoparticle surface functionalization. In this study, the capillary electrophoresis hyphenated with inductively coupled plasma tandem mass spectrometry (CE-ICP-MS/MS) was used for the first time to monitor gold–drug nanoconjugates formation. The research included optimizing CE separation conditions and determining reaction kinetics using the CE-ICP-MS/MS developed method. To characterize nanocarriers and portray changes in their physicochemical properties induced by the surface’s processes, additional hydrodynamic size and ζ-potential by dynamic light scattering (DLS) measurements were carried out. The examinations of three types of functionalized GNPs (GNP-PEG-COOH, GNP-PEG-OCH_3_, and GNP-PEG-biotin) distinguished the essential differences in drug binding efficiency and nanoconjugate stability.

## 1. Introduction

According to WHO data, cancer is the second most frequent cause of death [1]. Despite the significant advances in medicine over the past few years, chemotherapy, suffering many limitations, is one of the most common methods of cancer treatment. Even when the use of innovative approaches increases, first-generation chemical drugs (including cytotoxic agents, e.g., leading platinum-based anticancer drugs) are used in single or multiple drug regimens (with known already or innovative drugs combinations). The employment of various agents or therapies (combined therapy) improves the therapeutic effect in cancer treatment, but side effects (regarding nonspecific activity) intensify [2,3,4]. Along with observed drug resistance (innated, acquired, or even cross-resistance), those limitations pose a real challenge [5]. Nanomaterials (NMs) employment as selective drug carriers was proposed as a solution. The main advantages of NMs application in drug targeted delivery systems (DTDSs) are increased selectivity towards cancer cells, improved drug bioavailability and solubility, and the decreased impact of blood plasma proteins on the therapeutic agent (reduced clearance) [5,6]. According to many reports, for various medical purposes (including drug delivery), gold nanoparticles (GNPs) are the most often applied among metallic NMs due to their surface plasmon resonance (SPR), stability, biocompatibility, and nontoxicity.

Typically, DTDSs are composed of nanocarriers and active molecules. Numerous examples of DTDSs’ types can be found in the literature, with variation in the uses of NMs and manner of drug accumulation. In the case of metallic nanoparticles used as nanocarriers, the drug is attached to the prepared surface of NMs. In this aim, the surface is often modified by cleavable (e.g., polymeric) molecules terminated with functional groups (or compounds abundant in such moieties) serving as linkers that bind therapeutics.

The specific surface area and the susceptibility to its modification and functionalization of particular NMs determine their use as nanocarriers in DTDSs. This prominent feature crucially affects the effective drug binding and transportation to destination cells, which can be performed two-fold: by passive or active mechanisms. According to literature, GNPs can attach various ligands (such as drugs, oligonucleotides, peptides, or antibodies), making them an ideal vehicle in targeted delivery [7,8,9]. Furthermore, a good nanocarrier should ensure efficient drug loading without any leakage during transportation into cancerous targets and release in a controlled manner. These features promote more significant drug accumulation, increasing cytotoxicity, treatment efficacy, and side effect reduction [6]. To fulfill such roles, the nanocarrier requires proper preparation of its surface by attuned modifier’s molecules terminated with specific functional groups [10,11].

Surface modifiers and functionalization type applied in DTDSs should exhibit stability throughout the transportation, with a protective effect preventing metabolic drug transformations and the possibility of the drug moiety cleavage at the therapeutic target (cancer cells) [8,9]. In many attempts, as a modifier attached to GNPs, polyethylene glycol (PEG) is used (“PEGylation”) [11,12,13], which is approved by US Food and Drug Administration (FDA). An additional issue is the size of formed DTDSs, which matters for two reasons: (i) FDA demands nanoparticles (NPs) used as pharmaceutical drugs to be metabolized or excreted from the body regarding the potential toxicity of NMs; (ii) a DTDS’s size affects cellular uptake and clearance. Therefore, it should be optimal to reach the tumor via enhanced permeability and retention effect and rapid renal removal of GNPs smaller than 6 nm [8,14].

GNPs’ wide applications are strongly related to currently developed studies of photothermal and photodynamic therapies (PTT and PDT, respectively). PTT and PDT exploit prominent features of GNPs such as ease of synthesis and surface modification, stability, physiochemical properties, and especially their application in targeted delivery [15]. Additionally, depending on (*a.o.*) which the surface modification was used, the efficacy of PTT and PDT varied. The combination and simultaneous activation of possible GNP mechanisms of action (i.e., anticancer drug delivery, photothermal effect, and reactive oxygen species generation) is challenging but can eradicate tumors highly efficiently.

**Figure 1:**
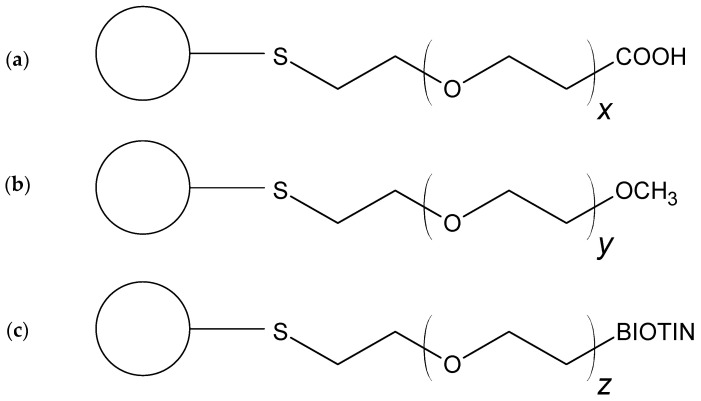
Three types of gold nanoparticle (GNP) functionalization used in gold nanoparticle–cisplatin system (GNCS) samples preparation: (**a**) GNP-PEG-COOH, polyethylene glycol (PEG) molecular weight of 3 kDa; (**b**) GNP-PEG-OCH_3_, PEG molecular weight of 2 kDa; and (**c**) GNP-PEG-biotin, PEG molecular weight of 5 kDa.

Considering described critical factors concerning DTDSs’ formation and the lack of comprehensive studies in this field, we decided to investigate the efficiency of GNP-based cisplatin delivery systems formation (including reaction kinetics), as compared to the nanocarrier surface functionalization type. Cisplatin (*cis*-dichlorodiammine platinum(II), an anticancer drug) was chosen due to its high cytotoxic potential and frequent use in chemotherapy (40–80% of all treated patients). Its effective binding can overcome well-known side effects and improve the therapeutic effect [6]. Despite the broadly described multiple examples of GNP surface modification and the variety of gold nanoparticle–cisplatin systems’ (GNCSs) formation protocols, there is no research concerning the direct and quantitative comparison of various functionalization types of impact on such conjugates’ formation efficiency and stability. Only a few works can be found in the literature, which suggest using the modifiers terminated with carboxylic moieties for this purpose [16]. According to in vitro (cell culture) and in vivo (animal) studies, GNCSs with specified surface coatings were reported to exhibit an improved therapeutic effect on cancerous cells (compared to that of the cisplatin used alone) [11,16]. Based on those reports, the presented work focuses on assessing various GNP coatings’ impacts on cisplatin binding efficiency. In our research, the conducted examinations employ three types of commercially available GNPs differing the length of surface modifier (PEG) and terminal group type responsible for drug binding (carboxyl-, methoxyl-, and biotin; see Figure 1).

In turn, in assessing cisplatin binding efficiency to functionalized GNPs, the choice of an appropriate analytical technique is significant [6]. Such assays should ensure simultaneous and online analysis of the reaction mixture with high sensitivity (on the trace levels of analytes), as well as enable distinguishing forms of reagents and to monitor changes occurring during the reaction. Frequently proposed in DTDS surveys, microscopic or spectroscopic techniques (e.g., UV-Vis) do not fulfill these requirements. Applying hyphenated techniques based on separation module and inductively coupled plasma mass spectrometry (ICP-MS) detection appears to be a powerful tool in DTDS monitoring. However, such coupling, employing capillary electrophoresis (CE)—CE-ICP-MS, was employed till now only once in metallic DTDS investigation [17]. Herein, we present the continuation of CE-ICP-MS-based technique employment, currently utilizing the tandem mass spectrometer equipped with the collision/reaction cell (CRC), regarding prospective monitoring of GNCSs and human–serum (HS) interactions. 

## 2. Results and Discussion

### 2.1. Gold Nanoparticle–Cisplatin Systems Preparation

In a previous study [17], the research of GNCS formation with the 11-mercaptoundecanoic acid (MUA), serving as a functionalization agent (equipped with COOH functional group) between the nanocarrier and the drug, was presented. However, the main limitations in the study were low stability, low formation efficiency of GNCSs, and a complicated and hardly repeatable synthetic procedure. As a solution, a novel method for preparing the GNCS nanoconjugates was elaborated. It includes choosing the GNPs’ functionalization type, thereby enabling effective cisplatin binding, optimization of cisplatin solution preparation, and optimization of reagent ratios based on reaction kinetics.

It is reported that covalent interactions between NPs and modifier molecules, achieved by the thiol group (–SH), ensure increased stability of nanoconjugates (especially in physiological conditions) [16,18,19] and can affect the cellular uptake [20]. As a modifier of GNPs’ surfaces, PEG with the thiol anchor was chosen in this research. The hydrophilic properties of PEG increase metal complex solubility and provide steric hindrance of NPs, improving their colloidal stability, and therefore diminishing the interactions with HS biomolecules (especially serum proteins) [12,19]. As a result, PEGylation contributes to decreased immune recognition and prolongs the half-life circulation (which increases with PEG chain elongation) [8,21].

**Figure 2:**
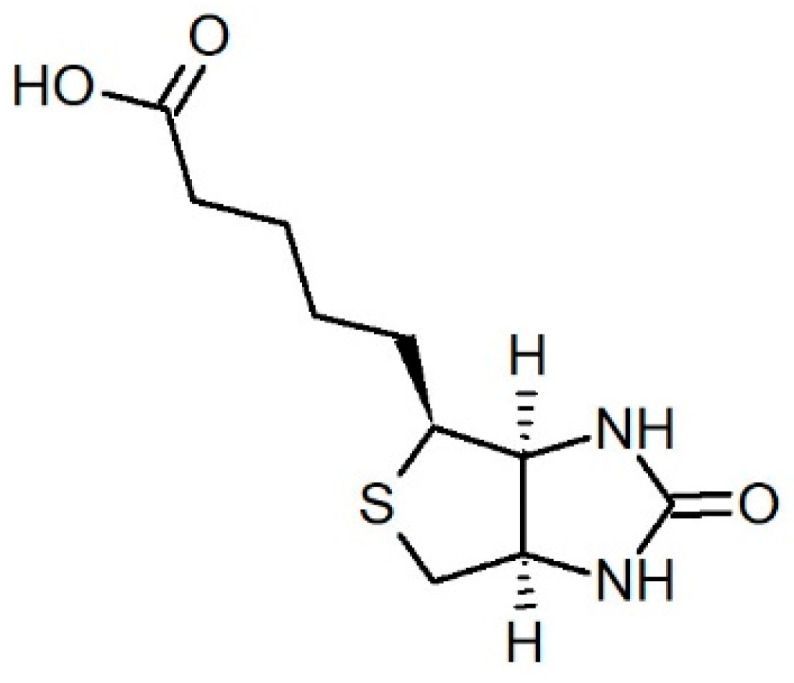
Biotin structure.

Another crucial issue is the proper form of the drug, which affects its binding efficiency significantly. In many surveys, the drug preparation for DTDSs includes so-called activation of the cisplatin intact form (CDDP) by AgNO_3_ addition, resulting in the exchange of Cl^−^ into more labile H_2_O ligands (cisplatin aquation). Obtaining two possible forms of the drug—[PtCl(H_2_O)(NH_3_)_2_]^+^ (intermediate) and [Pt(H_2_O)_2_(NH_3_)_2_]^2+^ (activated, CDDP*)—allows for the binding of the platinum atom (which is, therefore, more accessible) with nucleobases of DNA. Moreover, the CDDP* favors the coordination bonds formation (preferred in DTDSs formation) between the drug molecule and functional group set at the end of modifier molecule (PEG). Intact cisplatin can be electrostatically adsorbed, which can cause its fast release in media with high-ionic strength (such as biological fluids) [16]. The previously proposed methodology [17] was insufficient, since the cisplatin derivatization reaction equilibrium moved quickly toward substrates. To obtain relatively stable bonds in GNCSs, three types of GNP functionalization (GNPs were coated with PEG serving as a surface modifier) were tested (see Figure 1). The nanocarriers varied in the molecular weight of used polymer and its terminal (functional) group (carboxylic—GNP-PEG-COOH; methoxylic—GNP-PEG-OCH_3_; biotin (5-[(3*aS*,4*S*,6*aR*)-2-oxo-1,3,3*a*,4,6,6*a*-hexahydrothieno[3,4-d]imidazol-4-yl]pentanoic acid, Figure 2)—GNP-PEG-biotin).

**Figure 3:**
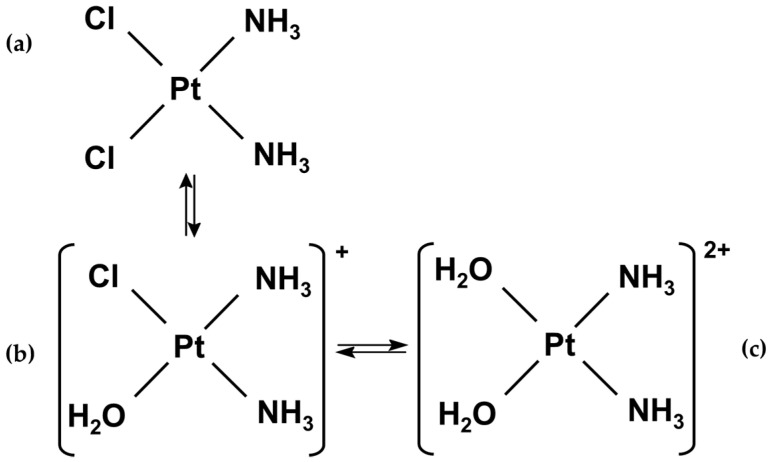
Scheme of reaction of cisplatin activation: (**a**) cisplatin intact form; (**b**) cisplatin intermediate form; (**c**) cisplatin activated (derivative) form.

Additionally, to attain better efficiency of cisplatin activation and greater derivative stability, the protocol was improved by a more extended time of the reaction run. The scheme of cisplatin equilibrium reactions is presented in Figure 3. The proposed methodology for CDDP* formation allows obtaining the solution with almost all molecules converted into [Pt(H_2_O)_2_(NH_3_)_2_]^2+^ and stable at time of incubation (12 h).

### 2.2. CE-ICP-MS/MS Method Figures of Merit

One of the initial steps of the research was the optimization of the CE-ICP-MS/MS method (used in the examination of formed DTDSs), which was performed to distinguish all sample constituents (not overreacted substrates and product). The obtained results are described in detail in Supplementary Materials and presented in Figures S1 and S2. The operational parameters of the optimized CE-ICP-MS/MS method are presented in Table 1.

The optimized CE-ICP-MS/MS method was evaluated regarding analytical parameters. The reproducibility of migration times (MTs) and areas of signals (relative standard deviation, RSD; *n* = 3) were calculated for all functionalized GNP types and cisplatin samples (intact and derivative) based on monitored isotopes signals (^197^Au^+^ and ^195^Pt^+^, respectively). Obtained values of reproducibility, the limit of detection (LOD), and the limit of quantification (LOQ) are presented in Table 2. Another studied parameter was the capillary recovery, which yielded 83% (an average of obtained values for particular substrates). The value was calculated by dividing the sum of the peaks’ areas corresponding to a specific analyte obtained during the analysis run under optimized conditions by the sum of the peaks’ areas obtained during the analysis conducted with applied 20 mbar of internal pressure.

### 2.3. Investigation of Gold Nanoparticle–Cisplatin Systems Formation, Reaction Kinetics, and Stability

**Figure 4:**
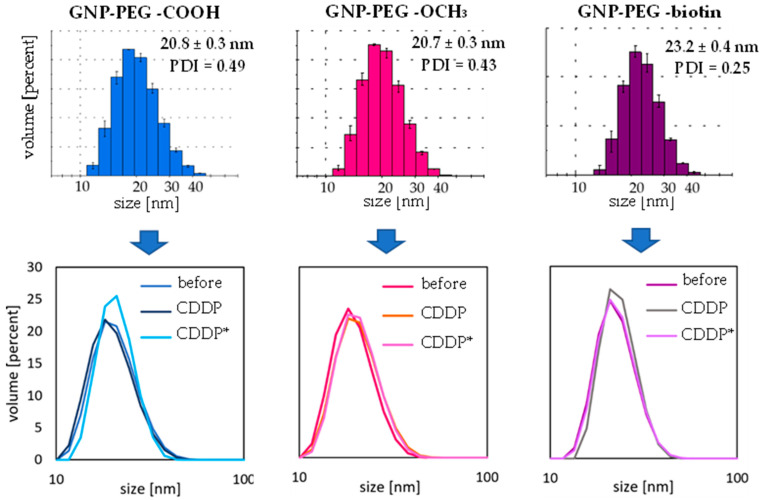
Values of hydrodynamic diameters (HDs) of gold nanoparticles (GNPs) and gold nanoparticle–cisplatin systems (GNCSs) obtained by means of dynamic light scattering (DLS). Top row presents histograms captured for each type of nanoparticle before modification (*n* = 3). Bottom row shows exemplary nanoparticle size distribution curves before and after modification with intact cisplatin (CDDP) and cisplatin derivative (CDDP*). HDs’ values for each modification were: GNP-PEG-COOH/CDDP (1:800)—21.3 ± 0.1 nm; GNP-PEG-COOH/CDDP* (1:800)—20.6 ± 0.4 nm; GNP-PEG-OCH_3_/CDDP (1:400)—20.0 ± 0.2 nm; GNP-PEG-OCH_3_/CDDP* (1:400)—20.5 ± 0.5 nm; GNP-PEG-biotin/CDDP (1:400)—23.5 ± 0.3 nm; GNP-PEG-biotin/CDDP* (1:400)—23.0 ± 0.3 nm.

To characterize the functionalized GNPs, used as nanocarriers, as well as to evaluate the influence of cisplatin and activated cisplatin adsorption on their colloidal stability and dispersion, a comparative analysis of GNPs’ and GNCSs’ hydrodynamic diameters (HDs) was carried out. The measured HDs of all analytes of interest exhibit the lack of significant differences related to the process of GNCS formation (small values of standard deviations confirm the monodispersity of all colloids). As shown in Figure 1, GNPs coated with various PEG ligands slightly differed in HD. The largest (23.2 ± 0.4 nm) was observed for PEG-biotin, and the smallest for PEG-OCH_3_ (20.7 ± 0.3 nm), which can be assigned to different lengths of PEG polymer molecules (5 kDa for biotin-terminated, 3 kDa for carboxy-terminated, and 2 kDa for methoxyl-terminated, respectively). All tested nanoparticles were characterized by a relatively small size dispersion (polydispersity index (PDI) of the nanoparticles before drug attachment does not exceed 0.49—see details in Figure 1). Moreover, with the presence of CDDP and its derivative in the mixture did not adversely affect the dispersion of GNCSs. As expected, HDs of GNPs turned out to be larger than the declared diameters of the metallic cores (10 nm). Such an increase in the HD is typical and can be explained by the bulky, polymeric character of the PEG (GNPs surface modificatory) and the different nature and spatial volumes of terminal functional groups [22]. The attachment of CDDP and CDDP* did not have a visible impact on the HDs of the obtained nanoconjugates, and it also did not induce their aggregation or loss of colloidal stability (see the bottom row in Figure 4). GNPs have high stability of their passivation combined with steric stabilization by bifunctional, thiolated-PEG ligands on the surface. A relatively thick, flexible, and highly hydrophilic PEG backbone is known to effectively protect NPs from the negative influence of the local environment [23].

**Figure 5:**
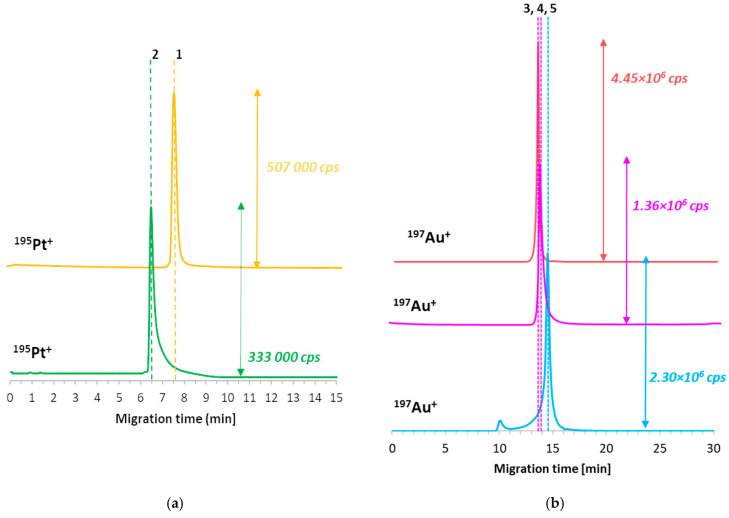
CE-ICP-MS/MS electropherograms of analyzed GNCS substrate samples: (**a**) CDDP (upper trace, signal no. 1) and CDDP* (lower trace, signal no. 2) in TB; (**b**) functionalized GNPs: with methoxylic moieties (upper trace, signal no. 3); with biotin as moieties (middle trace, signal no. 4); with carboxylic moieties (lower trace, signal no. 5) in TB. Separation conditions are described in Table 1.

The next step of the research was the evaluation of the GNCSs’ formation kinetics. In the beginning, MTs of all substrates were determined to (i) indicate particular sample components and (ii) assess substrates’ conversion ratios toward the final product (GNCSs). The optimized method enabled the separation of both considered cisplatin forms: derivative (6.53 min) and intact (7.50 min) (Figure 5a). CDDP* was registered on the electropherograms earlier due to the resultant positive charge of the molecule with substituted H_2_O ligands. The MTs of functionalized GNPs do not differ radically: 13.36 min for GNP-PEG-OCH_3_, 13.60 min for GNP-PEG-biotin, and 14.50 min for GNP-PEG-COOH (see Figure 5b). In the case of GNPs with carboxylic moieties, the additional signal near 10 min is visible; this signal probably corresponds to the aggregate. As further analyses indicated, this form of nanomaterial does not undergo the reaction with cisplatin.

**Figure 6:**
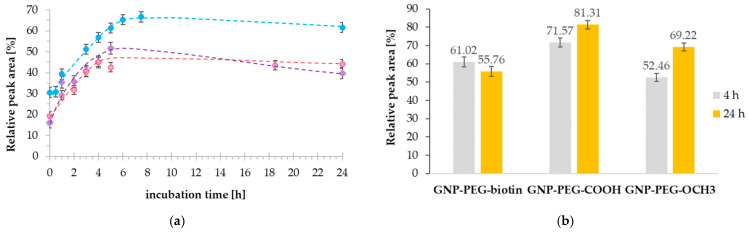
Reaction efficiency of GNCS formation (substrates ratio: 1 GNP per 800 cisplatin molecules) in time (37 °C, 400 rpm) expressed as relative peak area of cisplatin corresponding to formed GNCSs: (**a**) reaction mixture samples composed of CDDP* and GNP-PEG-COOH (blue dots), GNP-PEG-OCH_3_ (pink dots), GNP-PEG-biotin (purple diamonds); (**b**) reaction mixture samples composed of CDDP and functionalized GNPs after 4 h and 24 h of incubation (37 °C, 400 rpm).

To evaluate the impact of the GNPs’ functionalization types on the DTDSs’ formation kinetics, first, the investigation underwent mixture incubation time (37 °C, 400 rpm). The highest efficiency of the drug binding to a particular NM, assessed herein, was expressed as the relative peak area of cisplatin bounded in formed GNCSs. Relative peak area was calculated as a percentage ratio of ^195^Pt^+^ peak corresponding to the nanoconjugate, which referred to all ^195^Pt^+^-containing peaks’ areas in the particular reaction mixture. According to our previous protocol [17], samples were prepared by mixing the GNPs and cisplatin in the molecular ratio of 1:800 in tricine buffer (50 mM, pH 8.0; TB) [17], then incubated and analyzed after specified time intervals ranging from 0 to 24 h, including the daily throughput of CE-ICP-MS coupling. Several mixtures (of each NPs type) were prepared to assess the incubation time influence on reaction efficacy. Each of them was analyzed after another time. The mixtures after the same time of incubation were probed three times (to obtain three replications). Figure 6a summarizes the outcomes of the experiment. In parallel, GNCSs formed with intact cisplatin were analyzed (Figure 6b), and results after 4 and 24 h of incubation at 37 °C are presented.

Despite the apparent similarity of phases in the rapid increase and steady-state of all reactions of GNCS type formation (Figure 6a), efficiency values constitute a difference. The rapid growth phase of the attached CDDP* molecule’s number to the GNPs’ moieties lasts up to 4 h of incubation in GNP-PEG-OCH_3_ case, up to 5 h in GNP-PEG-biotin case, and up to 6 h in GNP-PEG-COOH case. Then, the efficiency slightly decreases and reaches the plateau state up to 24 h of incubation. Among all GNP types, the best drug binding efficiency was observed for GNP-PEG-COOH NMs, independent of the considered time and drug form. At the same time, GNPs with methoxyl- and biotin groups attached the drug less efficiently. The outcome suggests the significant impact of such GNP functionalization in DTDS formation. During incubation time longer than 4–6 h, the drug concentration is relatively constant, indicating that the formed GNCSs do not release drug molecules significantly (especially in the GNP-PEG-OCH_3_ case). Slight differences can be observed for GNCSs employing carboxyl- and biotin groups on GNPs’ surfaces. Considering reaction mixtures of GNPs with biotin groups (regarding CDDP* and CDDP use), the efficiency of the drug-binding was lower when the incubation time was extended. In comparison to GNCSs with -OCH_3_ and -COOH moieties, the results contribute to the conclusion that nanoconjugates based on GNP-PEG-biotin can feature decreased long-term stability (over 24 h of incubation).

The observed differences in drug binding efficiency can be related, on the one hand, to the slightly basic pH of reaction media (pH 8.0), which enables the activation (deprotonation) of some functional groups. According to obtained ζ-potential values (described in detail hereinafter), all considered nanoconjugate types are characterized with negative parameter values. The anionic character of those NMs arises from the intrinsic negative surface charge of GNPs [24], but also the deprotonated functional groups, especially carboxylic (in the case of GNP-PEG-COOH), favoring the CDDP* attachment [19]. On the other hand, in the GNP-PEG-biotin case, the possible steric hindrance caused by biotin molecules can impede the CDDP* access to donor areas and results in reaction time elongation (slower kinetics of drug attaching). Although the conducted analyses enabled the establishment of the best GNCSs formation time, these values were concerned. Considering all preparation procedures, including capillary conditioning, cisplatin activation, and then mixtures incubation for 4–6 h, it turned out that they are highly time-consuming, limiting the daily throughput of conducted analyses since the applied methodology cannot be automated. As a solution, the incubation time of mixtures was reduced to 4 h.

An additional issue in drug-binding efficiency comparison is the character of the drug and GNP interactions. In this aim, GNCSs formed with CDDP were probed regarding the specified times of the mixtures’ incubation—4 h (as the already attuned optimal reaction time) and 24 h (to compare the stability). The experiments (Figure 6b) showed this issue’s crucial impact on the drug binding. Depending on the process (reaction) conditions, type of terminal functional groups, and drug charge, the character of present herein noncovalent interactions between NMs and the drug is diverse. CDDP or CDDP* association to GNPs results from various interactions mechanisms (electrostatic, donor-acceptor, hydrogen bonds) occurring in changeable ratios. In the case of GNCSs formed with CDDP, the predominant electrostatic interactions appear to be more effective, resulting in increased efficiency (up to 81.31% after 24 h of incubation). Nevertheless, despite the promising outcomes, reported instability and weak character of those bonds [16] were confirmed in just preliminary tests of chosen GNCSs treated with HS. As a result, GNPs and CDDP* associations were used for further investigations (in which hydrogen or donor-acceptor bonds prevail).

**Figure 7:**
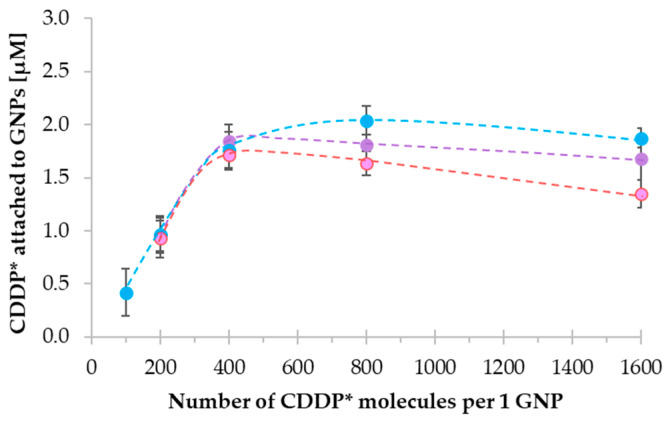
Reaction efficiency (*n* = 3) of GNCSs formation (expressed as molar concentration of CDDP* attached to GNPs) after 4 h of incubation (37 °C, 400 rpm) depended on ratio of substrates: CDDP* and GNP-PEG-COOH (blue dots), GNP-PEG-OCH_3_ (pink dots), and GNP-PEG-biotin (purple dots).

The next crucial step was establishing the reagent ratio in mixtures, resulting in the best efficacy of DTDS formation expressed in the molar concentration unit of attached cisplatin. The efficacy was calculated based on the area under the ^195^Pt^+^ peak (as a percentage of all ^195^Pt^+^-containing peaks’ areas) corresponding to formed GNCSs. In this aim, reagents were mixed in TB in ratios ranging from 200 to 1600 CDDP* molecules per 1 GNP (1:200, 1:1600, respectively), incubated for 4 h (37 °C, 400 rpm), and analyzed. Comenge et al.’s protocol [16] synthesized GNCSs by employing MUA-functionalization; the drug loading efficiency was calculated, and it yielded 470 cisplatin molecules per one nanoparticle (based on ICP-MS measurements). The authors also synthesized GNPs whose core sizes were more extensive than NPs used in current work (~13 nm vs. 10 nm). Therefore, we decided to test various molecular ratios of reagents and assess the drug loading efficiency. Obtained results are presented in Figure 7. The investigation indicated that the reaction efficiency and course depend on the applied drug concentration and vary regarding the used GNPs’ functionalization types. The most distinct impact of the ratio of the reagents can be observed for the GNP-PEG-COOH. The best efficiency of CDDP* loading (2.0 µM) was obtained in the case of reagents ratio of 1:800. In the case of GNPs terminated with methoxyl- and biotin moieties, the biggest efficiency values were registered for the 1:400 ratio. Based on the course of the discussed reactions (the phases of the rapid increase and plateau), those points correspond to the saturation state of active moieties on the GNPs’ surface. Simultaneously, the excess of the drug does not improve the reaction efficiency but contributes to the decrease in drug binding by GNPs. The decreased efficiency values at higher cisplatin concentration levels observed in GNP-PEG-COOH-based nanoconjugates can be related to the raising neutralization of -COOH groups reported by Tan et al. [19]. In the research concerning the CDDP*–GNPs systems that utilize dendron and ended with the carboxylic group, the authors describe the diminished stability of formed conjugates at higher CDDP* concentrations. The neutralization caused the weaker charge stabilization of the functionalized GNPs’ surfaces, resulting in reduced drug binding efficiency.

Another approach to assess the effectiveness of the formation of GNCSs, which employ various NMs’ surface functionalizations, is the comparison of the number of linker moieties on the GNPs’ surfaces that attached CDDP*. The calculations were performed based on the producer specification (the estimated density of moieties per 1 nm^2^ of GNP) and obtained results related to the concentration of the drug attached to GNPs in the time of 4 h of incubation (Table 3). The research discussed three types of GNP functionalization that vary the number of PEG monomers and moiety able to bind the drug. Therefore, the significant role in individual GNCS formation has a size of steric hindrance of the linker, determining the number of active regions accessible for the drug binding. Among the considered types of GNP functionalization, the lowest density of the functionalizing agents on the NM’s surface was estimated in the case of GNP-PEG-biotin, which suggests a decreased (compared to other) number of binding sites. In contrast to those assumptions, the saturation efficiency of accessible moieties with CDDP* reached almost 40%. The value was much higher than for other tested NMs, especially GNP-PEG-OCH_3_. Despite the high steric availability and the wealth of binding sites, CDDP* demonstrated the lowest affinity to -OCH_3_ terminal groups in this case.

As a result of the study, the best optimal reaction efficacies were obtained after 4 h of incubation and at the ratio of the following reagents: 1:400 for GNP-PEG-OCH_3_/CDDP* and GNP-PEG-biotin/CDDP* samples, and 1:800 for GNP-PEG-COOH/CDDP*. The first approach in GNCSs colloids investigation were DLS studies including HD, particle size distribution, stability (as mentioned above), and ζ-potential measurements. The measured values of ζ-potential, as obtained by GNPs and GNCSs in TB, are summarized in Table 4. The values of this parameter vary from −19.9 to −27.1 mV in all GNPs and DTDSs types and do not suggest any significant changes in the charge stabilization on the NMs’ surfaces, resulting from performed GNCS synthesis. All samples distinguish an anionic character, represented by negative ζ-potential values, which is typical for metallic nanoparticles without ligands of well-defined, positive charge [21]. Importantly, results did not show significant changes in the surface charge induced by drug molecule association, even though CDDP* gains a positive charge after losing chloride anions. However, due to the relatively low surface densities of the associated CDDP and CDDP* molecules and the protective nature of PEG polymer, their influence on the GNCSs surface charge investigated using the DLS technique turned out to be negligible. The relatively high ionic strength of the surrounding medium (TB) is also essential, which results in the charge screening and thus suppresses changes in charge of GNP-drug nanoconjugates.

**Figure 8:**
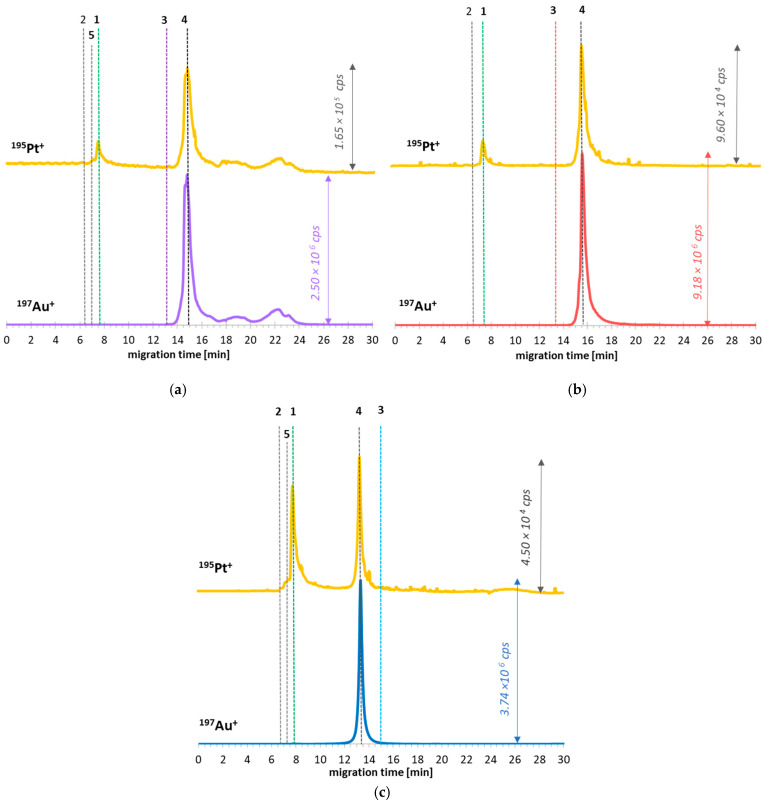
CE-ICP-MS/MS electropherograms of GNCS formation in optimized reaction conditions: (**a**) GNP-PEG-biotin/CDDP* systems in TB (tricine buffer, 50 mM, pH 8.0) (4 h of incubation, 37 °C, 400 rpm, 1:400 ratio); (**b**) GNP-PEG-OCH_3_/CDDP* systems in TB (4 h of incubation, 37 °C, 400 rpm, 1:400 ratio); (**c**) GNP-PEG-COOH/CDDP* systems in TB (4 h of incubation, 37 °C, 400 rpm, 1:800 ratio). Separation conditions are described in Table 1. Signals assignment: **1**—CDDP; **2**—CDDP*; **3**—respective functionalized GNPs (substrate); **4**—respective GNSCs; **5**—cisplatin intermediate form.

The electropherograms of such prepared samples are presented in Figure 8. The newly formed species of ^197^Au^+^ and ^195^Pt^+^ were registered on electropherograms (signal no. 4), corresponding to formed nanoconjugates. In all these cases, the migration time (MT) of ^197^Au-containing peaks differs from the MT of GNP substrates, confirming the formation of GNCSs. Moreover, the vanishing of CDDP* peak (no. 2) in all cases was observed, while small peaks of intact cisplatin appeared. Additionally, in Figure 8a,c, the newly not intense peak is visible at the MT placed between CDDP and CDDP* signals (no. 5), suggesting the formation of the intermediate cisplatin form. The presence of CDDP and intermediate drug form confirms that the reaction equilibrium was partially moved into the substrates. In parallel, the rest of the CDDP* molecules present in the mixture underwent conversion into GNCSs. Furthermore, intense and broad peaks with irregular shape in ^195^Pt^+^ and ^197^Au^+^ traces of GNP-PEG-biotin/CDDP* sample electropherogram (Figure 8a) point to the formation of aggregates/agglomerates, which still can attach the activated drug.

Complementarily, UV-Vis measurements of discussed functionalized GNPs and GNCSs were performed. Studies on absorption spectra are often applied in NMs investigations on the size, shape, aggregation, and synthesis reaction monitoring via observation of the changes in SPR shifts and peaks intensities [25]. In the current study, UV-Vis spectroscopy was used to monitor the possible changes in size and colloidal stability of formed GNCSs after various incubation times (4 h—elaborated as the optimal time for the best binding efficiency obtaining; 24 h and 48 h to assess the stability) (Figure S3).

In all UV-Vis spectra of GNPs, the single, quite slim, and intense peaks were registered with the maximal absorbance at wavelength 520 nm, which confirmed the small size, spherical morphology, and monodispersity of spherical nanoparticles [26]. The shifts of SPR peaks were not observed, suggesting the absence of aggregates forming along with the extending time of samples incubation, consistent with the earlier DLS results. The slight differences in the maximal absorbance values can be seen between the samples employing CDDP and CDDP*. GNCSs samples also did not exhibit increased scattering (represented by the high baseline absorbance at long wavelengths) to the corresponding GNPs. The only exception is GNP-PEG-biotin/CDDP; however, this effect was insignificant. This also proves a good dispersion of the obtained GNCSs and their negligible aggregation.

As follows from the abovementioned outcomes, DLS, ζ-potential, and UV-Vis analyses provide essential information on nanoconjugates’ size, morphology, and stability. However, they are insufficient to provide qualitative information on DTDS formation efficiency related to the drug. Such results can be due to the small size of the cisplatin molecule, which does not significantly affect the size of formed GNCSs. Additionally, cisplatin does not absorb UV-Vis radiation and remains in equilibrium with its discussed forms; thus, its impact in such analyses cannot be directly assessed.

## 3. Materials and Methods

### 3.1. Chemicals

Elix Water Purification system (Merck Millipore, Molsheim, France) was used to obtain the high purity water (R ≥ 15 MΩ cm^−1^) to prepare all the aqueous solutions. Spherical GNPs (colloidal stock solutions, 10 nm core size, 2.99 × 10^14^ NPs mL^−1^) with various surface coatings (methoxyl-PEG2000-SH-GNPs, carboxyl-PEG3000-SH-GNPs, biotin-PEG5000-GNPs) were purchased from Cytodiagnostics (Burlington, ON, Canada). Cisplatin (*cis*-diammineplatinum (II) dichloride, ≥99.9%), silver nitrate, tricine, sodium phosphate monobasic, ammonium hydrogen carbonate, Trisma^®^ base (2-amino-2-(hydroxymethyl)-1,3-propanediol), with purity ≥99%, and sodium phosphate dibasic (≥98,5%) were purchased from Sigma–Aldrich (St. Louis, MO, USA). Sodium hydroxide (HPLC purity) and germanium ICP standard solution (1 g L^−1^) were purchased from Supelco (Bellefonte, PA, USA), and acetic acid (HPLC purity) from Sigma–Aldrich (Darmstadt, Germany). Methanol (LC-MS grade) was purchased from POCH (Gliwice, Poland). Gases (argon and oxygen) of purity ≥99.999% (Messer, Bad Soden, Germany) were used for CRC in ICP-MS/MS.

### 3.2. Nanocarrier–Drug Systems Preparation

In this research, GNCSs with three types of GNPs’ surface functionalization that vary used moieties (methoxyl-, carboxyl-, and biotin-GNPs) were investigated. Each GNCSs type was prepared two-fold (to assess the efficiency of drug binding and stability of formed nanoconjugates): once with the use of aqueous CDDP (intact) solution (100 µM), and once with the use of cisplatin derivative solution (100 µM) (CDDP*). GNCS samples were prepared in TB (50 mM, pH stabilized with NaOH 1 M solution up to the value of 8.0) by mixing functionalized GNPs and CDDP or CDDP* solution in varying reagent ratios (from 200 to 1600 drug molecules per 1 gold nanoparticle) and varying incubation times (0–24 h). Samples were incubated at 37 °C with stirring (400 rpm) in a MultiTherm incubator (Benchmark, Lodi , NJ, USA). The choice of proper concentrations (and sample volume injected to CE capillary) for both GNPs and cisplatin analytes was significant. Calculations based on proposed ratios indicated the high GNP and low drug concentrations, which on the one hand can cause the decrease in capillary recovery, and on the other hand cause the difficulty in ^195^Pt^+^ signals quantification. The last issue is related to low final cisplatin concentrations in samples close to LOQ value, resulting in decreased signal-to-noise ratio (S/N), especially in the samples of 1:200 molecular ratio. According to optimized sample volume, the final applied concentration of GNPs was 3.74 × 10^12^ NPs mL^−1^ (which equals 0.38 mg mL^−1^ of ^197^Au), and cisplatin ranged from 1.25 to 15 µM.

CDDP stock solution was prepared by dissolving 1 mg weight in 2 mL water in an ultrasound bath (Bandelin Sonorex DT 52 H, Berlin, Germany) and incubating in the dark for up to 2 h because of the drug hydrolysis process. New CDDP intermediate solutions (100 µM) were prepared before mixing with GNPs. CDDP* solution preparation was carried out by the revised methodologies described in the literature [19,27]. The derivative (compared to that of the intact form) characterizes the substitution of Cl^−^ by H_2_O ligands, which enhances the molecule reactivity. Briefly, the weighted amounts of CDDP (15 mg) and AgNO_3_ (16.9 mg) were dissolved in 2.5 mL of water with the ultrasound bath utility and then mixed. The solution was incubated in the dark for 3 h at 60 °C (400 rpm) and overnight at 22 °C (400 rpm) (MultiTherm incubator, Benchmark, Lodi , NJ, USA). Then, the solution was purified from AgCl residues in two steps: (1) centrifugation (30 min, 12 000 rpm, MPW-352RH centrifuge, Poland) and (2) double filtration with syringe filters (equipped with the polytetrafluoroethylene membrane, 0.2 µm pore size, ChemLand, Stargard, Poland). A new CDDP* stock solution was prepared each day and used (maximally within 12 h) for samples preparation. New CDDP* intermediate solutions (100 µM) were prepared before mixing with GNPs. In CE-ICP-MS/MS and UV-Vis measurements, the GNP-CDDP and GNP-CDDP* associate samples were prepared similarly (the same concentrations of particular colloidal solutions were applied). For DLS and ζ-potential measurements, all samples were diluted twice in TB. To calculate the figures of merit in the optimized CE-ICP-MS/MS method, the samples of GNPs and cisplatin (intact and derivative) in TB were analyzed (concentrations of 3.74 × 10^12^ NPs mL^−1^ and 30 µM, respectively) at least three times.

### 3.3. CE-ICP-MS/MS Instrumentation

Analyses were performed using the CE-ICP-MS/MS hyphenation: a 7100 CE system (Agilent Technologies, Waldbronn, Germany) coupled to an 8900 ICP tandem mass spectrometer (Agilent Technologies, Santa Clara, CA, USA) working in MS/MS mode using collision/reaction gases. Polyimide-coated fused silica capillaries (i.d. 75 µm, o.d. 375 µm, length 70 cm) were purchased from C&M Scientific Ltd. (Silsden, UK). The sample introduction system was based on the CEI-100 nebulizer interface (Teledyne CETAC Technologies, USA) equipped with a low-volume spray chamber and a cross-piece merging the sheath liquid flow (10 ppb ^72^Ge solution in phosphate buffer 1 M solution). The CE electrical circuit was performed via a grounded platinum wire. The constant sheath liquid flow (closing the electrical circuit) was kept thanks to a self-aspiration mode of the nebulizer working. The capillary cassette and sample tray temperatures were held at 37 °C by a thermostat. Instrument control and analysis of collected data were performed by Agilent MassHunter Workstation and Agilent ChemStation software utility.

Newly used capillaries were activated by rinsing with the 1 M NaOH solution (40 min) and H_2_O (30 min). Each day (before use), the capillary was conditioned with 1 M NaOH (20 min), then with H_2_O (15 min), and a background electrolyte (BGE) (15 min). Between analyses, the capillary was flushed in the following order: 1 M NaOH (1 min), a mixture of 1 M NaOH, methanol, and water (25/50/25, *v*/*v*/*v*) (1 min), H_2_O (1 min), and BGE (2 min). The manner enabled the memory effect elimination and capillary lifetime elongation. ICP-MS was tuned by a standard solution of ^7^Li, ^59^Co, ^89^Y, ^140^Ce, ^205^Tl (concentration of 10 ng mL^−1^ each, in 2% HNO_3_) to obtain the highest signals for yttrium and cobalt isotopes and the lowest levels of polyatomic, oxide, and doubly charged ions.

As an internal standard in the sheath liquid for the CE-ICP-MS/MS, a 10 ng mL^−1^ germanium (^72^Ge) solution was used. The monitoring of ^72^Ge^+^ during CE-ICP-MS analyses and after the capillary rinsing was crucial in controlling the hyphenation stability and nebulization efficiency. Germanium does not suffer interferences in applied analysis conditions. Every investigation was initiated when the ^72^Ge^+^ à ^72^Ge^+^ signal was sufficiently stable (RSD, <2%) and high (counts per second, cps, >5000).

### 3.4. UV-Vis Measurement

UV-Vis absorption spectra were recorded for functionalized GNPs’ colloidal solutions and gold nanoparticle–cisplatin nanoconjugates (CDDP and CDDP* variants in TB). Analyzed GNCSs samples were prepared in various (optimized) molecular ratios of reagents (nanomaterial to drug molecules) and incubated for 4 h, 24 h, and 48 h at 37 °C with stirring (400 rpm) before the measurement. The preliminary purification process of the reaction mixture from the drug excess was not required since cisplatin molecules do not exhibit absorption in the studied spectral range. As a control probe, the corresponding functionalized GNP samples were used (as a background sample, TB was used). Spectra were recorded at the wavelength ranging from 350 to 800 nm (a resolution of 1 nm) in 2 mL glass cuvettes using Jasco V-730 Spectrophotometer (Tokio, Japan).

### 3.5. DLS and ζ-Potential Measurement

The measurements of HD and ζ-potential underwent the samples of functionalized GNPs and GNCSs (both CDDP and CDDP* variants). GNCS samples were prepared in optimized reagents ratios and concentrations, incubated for 4 h (at 37 °C with stirring, 400 rpm), and diluted twice with TB (NMs’ final concentration—1.87 × 10^12^ NPs mL^−1^) before the analysis. All measurements were conducted at 25 °C after 60 s of temperature stabilization using Zetasizer Nano ZS (Malvern Panalytical, Malvern, UK). The excitation wavelength of the DLS instrument was 633 nm (He-Ne laser, power = 5 W), and the measurement angle was 173°. Disposable cuvettes made of polystyrene and dip cell equipped with palladium electrodes (Malvern) were used during hydrodynamic diameter and ζ-potential measurements, respectively. All DLS and ζ-potential measurements were conducted with at least three replications.

## 4. Conclusions

In the research, the impact of the gold nanoparticle surface functionalization type on the yield of cisplatin targeted delivery systems formation was evaluated, which was not investigated until now, despite the variety of such systems’ formation protocols. Significant differences in cisplatin binding efficiencies were observed among discussed gold nanoparticles (GNPs) that varied the surface coatings (polyethylene glycol (PEG) terminated with -COOH, -OCH_3_, and biotin moieties).

The obtained results confirmed that the type of functionalization of the used nanocarrier determines its ability to bind cisplatin effectively. Depending on the used moiety, *a.o.*, its steric hindrance, time of conducted reaction, and the pH of the reaction mixture media, the concentration and form of the drug can crucially impact the formation and long-term stability (including drug release and colloidal stability) of gold nanoparticle–cisplatin systems (GNCSs). The moiety charge stabilization and accessibility of binding sites are significant factors. Obtained results indicated the highest affinity of the drug to GNPs covered with PEG-COOH. Formed nanoconjugates feature the highest stability over incubation (24 h), resulting in minimal drug release. Moreover, the investigations revealed the differences in the kinetics of the particular GNCS formation reactions. The results provided herein constitute the prerogative for further studies in this field. Collected data could contribute to developing drug targeted delivery systems (DTDSs) for medical purposes, with improved drug loading efficiency and stability properties, achieved thanks to attuned linkers between the nanocarrier and drug.

In conducted work, few techniques were used to characterize formed DTDS, and their usefulness was confirmed. However, the most comprehensive turned out to be the CE-ICP-MS/MS technique, which is indicated as a method of choice in such examinations (which was suggested previously). In contrast to performed UV-Vis and DLS/ ζ-potential measurements, CE-ICP-MS/MS technique provides clear qualitative and quantitative information of GNCS formation and stability, thanks to the possibility of each sample component’s simultaneous separation and isotopically specific identification with low limits of detection (LODs).

## Figures and Tables

**Table 1:** Optimized CE-ICP-MS/MS method’s operational parameters.

**CE System**	
Capillary	Fused silica capillary, i.d. 75 µm, o.d. 375 µm, 70 cm length
BGE *	Phosphate buffer, 10 mM, pH 7.4
Temperature	37 °C
Voltage	+17 kV
Current	20–40 µA
Sample injection	Hydrodynamic, 30 mbar, 5 s
**ICP-MS/MS System**	
RF power	1570 W
Sample depth	8.2 mm
Plasma gas	15.0 L min^−1^
Nebulizer gas flow	1.10 L min^−1^
Sheath liquid	10 ng mL^−1 72^Ge in 1 mM PB
Sheath liquid flow	10 µL min^−1^
Cell gas (O_2_) flow	0.51 mL min^−1^
Monitored mases	^72^Ge^+^, ^197^Au^+^, ^195^Pt^+^, ^32^S^16^O^+^

* BGE—background electrolyte.

**Table 2:** Figures of merit of optimized CE-ICP-MS/MS method.

Analyte	RSD (%) (*n* = 3)		LOD (mol L^−1^)		LOQ (mol L^−1^)	
	Migration Time	Peak Area	^195^Pt	^197^Au	^195^Pt	^197^Au
CDDP	5.1	7.0	2.16 × 10^−8^	–	6.47 × 10^−8^	–
CDDP*	2.7	7.5	3.87 × 10^−8^	–	1.16 × 10^−8^	–
GNP-PEG-biotin	0.8	2.8	–	2.29 × 10^−11^	–	6.88 × 10^−11^
GNP-PEG-OCH_3_	8.9	4.7	–	8.59 × 10^−12^	–	22.58 × 10^−11^
GNP-PEG-COOH	0.7	8.7	–	7.17 × 10^−12^	–	2.15 × 10^−11^

**Table 3:** Efficiency of drug binding to functionalized GNPs in dependence of linker moieties density on GNPs surfaces.

GNPs Functionalization Type (Terminal Moiety); Reagents Ratio **	The Estimated Density of PEG-Moieties on the GNPs Surface	The Estimated Number of PEG-Moieties on the Surface of Each GNP [-]	The Highest CDDP* Concentration in GNCSs [µM]	Efficiency of Moieties Saturation with CDDP* Molecules [%]
Methoxyl-; 1:400	4 per 1 nm^2^	~1256	1.77	4.55
Carboxyl-; 1:800	1 per 1 nm^2^	~314	2.12	21.70
Biotin; 1:400	0.5 per 1 nm^2^	~157	1.91	39.23

** Best molecular ratio of reagents results in greatest concentration of drug attached to nanomaterial in individual GNCSs.

**Table 4:** ζ-potential values recorded for GNP samples before and after CDDP and CDDP* conjugation (*n* = 3).

GNPs Functionalization (Terminal Moiety); Reagents Ratio **	before [mV]	CDDP [mV]	CDDP* [mV]
Methoxyl-; 1:400	−24.6 ±1.0	−24.1 ±1.0	−24.3 ±1.3
Carboxyl-; 1:800	−21.4 ±1.0	−23.1 ±1.8	−27.1 ±0.8
Biotin; 1:400	−19.9 ±1.2	−19.4 ±0.7	−22.7 ±0.8

** Best molecular ratio of reagents results in greatest concentration of drug attached to nanomaterial in individual GNCSs.

## Data Availability

Data available on request due to restrictions, e.g., privacy or ethical. The data presented in this study are available on request from the corresponding author.

## Supplementary Materials

The following supporting information can be downloaded at https://www.mdpi.com/article/10.3390/ijms23042324/s1. References [28,29,30,31,32,33] are cited in the supplementary materials.
