# Supplementary material for: Targeted Delivery of Cisplatin by Gold Nanoparticles: The Influence of Nanocarrier Surface Modification Type on the Efficiency of Drug Binding Examined by CE-ICP-MS/MS
Source: Int J Mol Sci. 2022 Feb 19;23(4):2324. doi: 10.3390/ijms23042324 (PMC8874575; doi:10.3390/ijms23042324)
Supplement: Supplementary file 1 [file ijms-23-02324-s001.zip › ijms-1600478-supplementary.pdf]

International Journal of *Molecular Sciences*

MDPI

Electronic Supplementary Information

Targeted Delivery of Cisplatin by Gold Nanoparticles: the Influence of Nanocarrier Surface Modification Type on the Efficiency of Drug Binding Examined by CE-ICP-MS/MS

Anna M. Wróblewska [1], Aleksandra Milewska [1], Marcin Drozd [2,3] and Magdalena Matczuk [1,*]

1 Chair of Analytical Chemistry, Faculty of Chemistry, Warsaw University of Technology, Noakowskiego St. 3, 00-664 Warsaw, Poland; awroblewska@ch.pw.edu.pl (A.M.W.); aleksandra.milewska2.stud@pw.edu.pl (A.M.)

2 Chair of Medical Biotechnology, Faculty of Chemistry, Warsaw University of Technology, Noakowskiego St. 3, 00-664 Warsaw, Poland; mdrozd@ch.pw.edu.pl

3 Center for Advanced Materials and Technologies, Warsaw University of Technology, Poleczki St. 19, 02-822 Warsaw, Poland

* Correspondence: mmatczuk@ch.pw.edu.pl; Tel.: +48-222-347-719

2. S. Results and Discussion

The evaluation of oxygen as a reaction gas in collision/reaction cell and optimization of its flow rate

Frequently, the ICP-MS analyses suffer the limitations of spectral (polyatomic and isobaric) interferences sourced by the sample matrix, reagents used for the preparation, entrained atmospheric gases, and plasma gases [1]. Mainly the phenomenon is observed in complex samples (primarily biological: human serum (HS), blood). Regarding the prospective investigations of GNCSs in simulated physiological conditions, it is crucial to study GNCSs–proteins interactions. In ICP-MS measurements, it is considered that the total protein abundance is correlated with the sulfur concentration, which is a building block of cysteine and methionine present in HS proteins. Therefore sulfur acts as a marker for those biomolecules in ICP-MS investigations [2]. Due to the reason that the ionization process of analytes in ICP-MS is performed in conditions of atmospheric pressure and air access, the significant interference originates from $^{16}O_2^+$. As a result, the measurements of $^{32}S^+$ suffer the interferences – the mass-to-charge ratios of $^{16}O_2^+$ and $^{32}S^+$ are equal (m/z=32) [3]. The problem can be overcome by the utilization of the ICP-MS equipped with the collision/reaction cell (CRC) system placed between two quadrupoles (MS/MS mode) [4] with oxygen used as a reaction gas (delivered to the CRC with inert gas), which is recommended to improve the sulfur measurements accuracy [5]. In this solution, particular analytes can be determined by their oxide form since Q_1 constitutes a mass filter selecting ions ($^{32}S^+$, m/z=32) for further reaction with the oxygen, and Q_2 selects the m/z with the mass shift mode of +16 ($^{32}S^{16}O^+$, m/z=48) [6]. In such a solution, sulfur is converted and indicated as an oxide form (Figure S1~~S4~~), while ozone ($^{16}O_3^+$) interference is not observed. The $^{195}Pt^+$ and $^{197}Au^+$ did not show any interferences in conditions of conducted analyses ($^{195}Pt^+ \rightarrow {}^{195}Pt^+$, $^{197}Au^+ \rightarrow {}^{197}Au^+$).

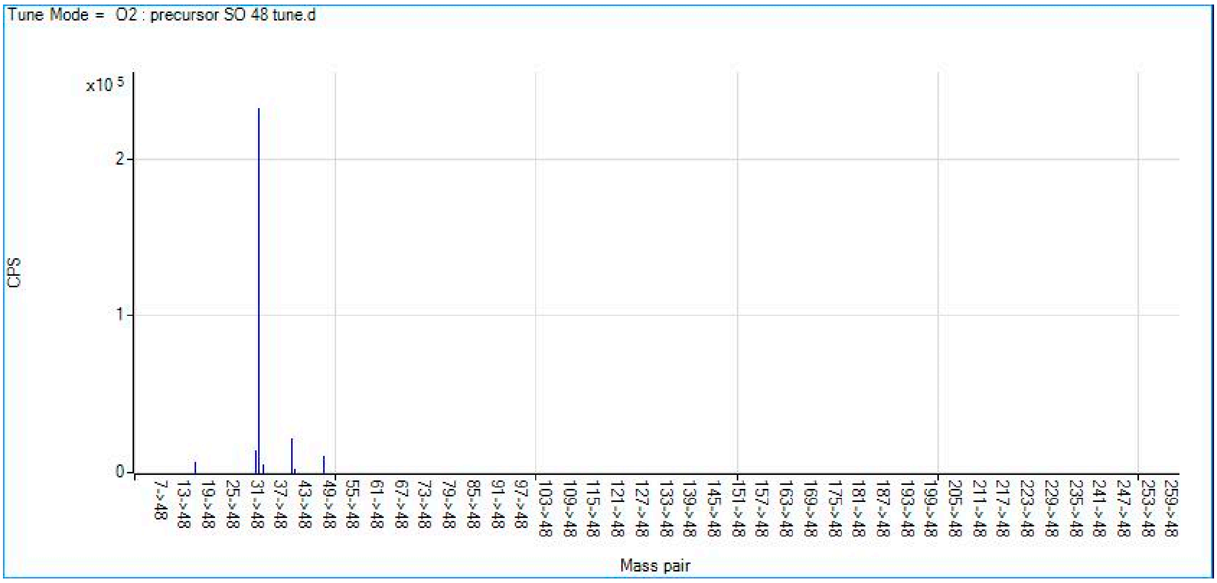

Figure S1. Precursor ion of *m*/*z* equal 48 ($^{32}S^{16}O^+$).

In this study, the reaction gas flow rate (O_2) was optimized to obtain the highest conversion rate of $^{32}S^+$ into $^{32}S^{16}O^+$ during CE-ICP-MS/MS analysis. It was elaborated based on the signal-to-noise ratio (S/N). The optimization was conducted using a sheath liquid containing a standards solution of ^{72}Ge (10 ng mL^{-1}), ^{197}Au (10 ng mL^{-1}), ^{195}Pt (10 ng mL^{-1}), and ^{32}S (100 ng mL^{-1}) with the increasing O_2 flow rate (from 0.27 mL min^{-1} to 0.60 mL min^{-1}). As a control solution, the sheath liquid containing only ^{72}Ge standard solution (10 ng mL^{-1}) was used. Oxygen flow at 0.51 mL min^{-1} was chosen since it ensured the compromised values of the conversion rate of $^{32}S^+$ and sensitivity towards $^{72}Ge^+$, $^{195}Pt^+$, and $^{197}Au^+$ analytes (Figure S2).

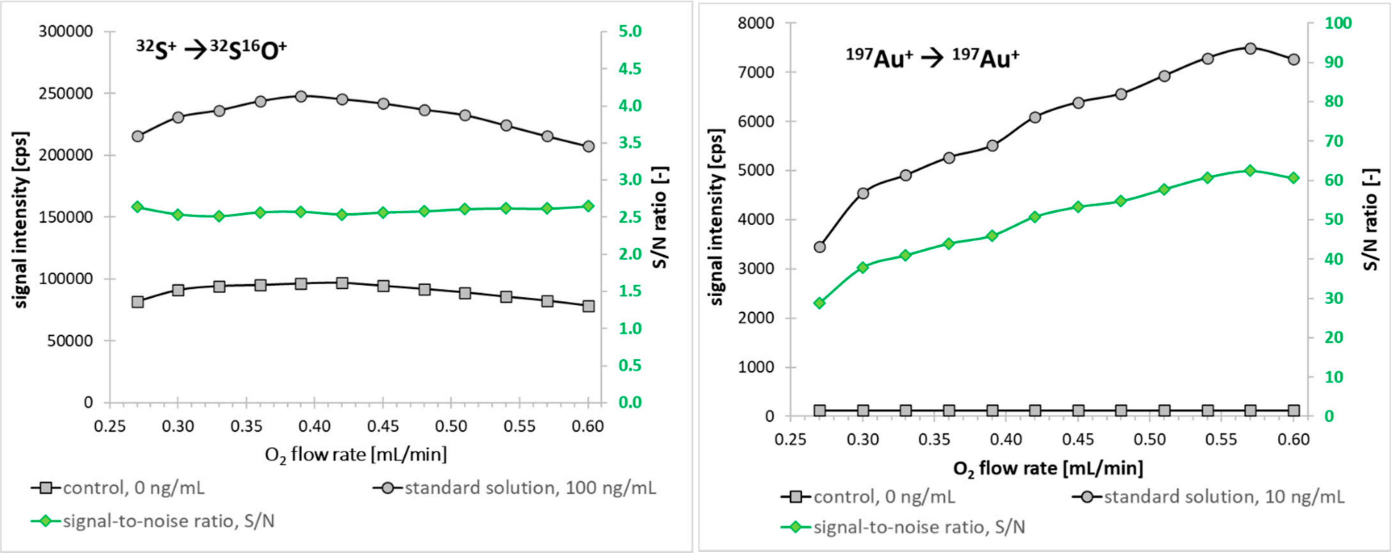

(**a**) (**b**)

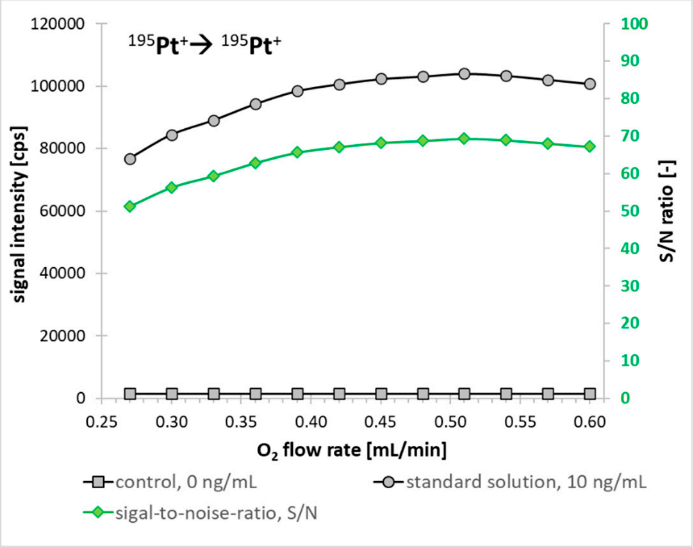

(**c**)

Figure S2. The evaluation of the reaction gas flow rate shown as the signal intensity and signal-to-noise (S/N) ratio dependence on the oxygen flow in the case of *m*/*z* (mass shift) equal: **(a)** 32→48 ($^{32}S^+$→ $^{32}S^{16}O^+$); **(b)** 195→195 ($^{195}Pt^+$→$^{195}Pt^+$); **(c)** 197→197 ($^{197}Au^+$→$^{197}Au^+$).

CE separation conditions optimization

To distinguish all sample constituents (not overreacted substrates and products), the optimization of CE separation conditions is required. To attain the best separation efficiency and resolution, the highest sum of peaks area, and the best Au and Pt recovery from the capillary, the evaluation of the (i) background electrolyte (BGE) type and (ii) its concentration, (iii) applied voltage value, and (iv) sample volume (injection) was performed.

The resolution was calculated based on the following equation (1), where t_1, t_2, and W_1, W_2 correspond to neighboring peaks (1, 2) migration times and peaks width at the base, respectively:

$$R = 2(t_2 - t_1)/(W_1 + W_2) \quad (1)$$

Regarding prospective investigations of formed GNCSs in human serum conditions (proteins interactions), electrolytes used as BGE should feature a marked buffering capacity close to physiological pH (*i.e.*, pH 7.4) to mimic the physiological conditions of the human blood. Additionally, their chemical structure should not contain sulfur or its impurities. For that reason, ultrapure ammonium hydrogen carbonate (AHC), phosphate buffer (PB), and Trisma® base (Tris) were proposed. Preliminary examinations of 5 mM solutions with a pH value at 7.4 were carried out on the cisplatin samples in which activated, intermediate and intact forms were present in changing ratios. The tested electrolytes showed the capability to distinguish a minimum of two of three cisplatin individuals initially. In the case of Tris, three signals were registered, however peaks intensity was low. In PB and AHC cases, signals were higher and more intense, but the resolution was lower (two signals were registered). Additionally, in the case of AHC, the current stability was disrupted, causing nonpersistent sheath liquid flow. The comparison of proposed electrolytes properties was conducted based on analyses of GNCSs samples (employing GNPs covered with PEG molecules terminated by biotin~~functionalization~~), indicating the BGE influence on the nanocarrier–drug system stability. Compared to PB, registered electropherograms for AHC and Tris did not reveal any signal corresponding to GNCSs, suggesting their negative impact on formed nanoconjugates~~GNCSs~~. As a result, the PB was used for further optimization conducted on GNCSs samples. The PB concentration effect on the area of the peaks was examined in the range of 5–15 mM, regarding the maximal

current amperage of 50 μA, which can be used in CE-ICP-MS hyphenation. The most extensive area under the peaks was achieved for 10 mM PB.

The following optimized operational parameter was the applied voltage that affects migration times and resolution. Among evaluated values (15-25 kV), the optimal separation ability of the CE-ICP-MS system was found at 17 kV, ensuring the initial current of *c.a.* 20 μA. Higher voltage values resulted in the resolution decrease which was caused by generating the current amperage of 40 μA. The last optimized parameter was the sample volume, enabling the highest signals to be achieved without impairing the capillary recovery. Due to the ratio of the applied reagents (high GNPs and low drug concentrations), the best sample loading was 30 mbar × 5 s. The injected to the capillary sample volume was enough to $^{195}Pt^{+}$ signals integration and did not cause the capillary clogging by GNPs.

UV-Vis, DLS, and ζ-potential measurement

In UV-Vis studies, each type of functionalized GNPs was mixed with two types of cisplatin solution (intact and derivative) at designated ratios: 1:400 in the case of GNP-PEG-biotin and GNP-PEG-OCH_3 (5 μM of cisplatin, 3.74×10^{12} GNPs mL^{-1}) and 1:800 in the case of GNP-PEG-COOH (10 μM of cisplatin, 3.74×10^{12} GNPs mL^{-1}).

In DLS and ζ-potential investigations, the samples of functionalized GNPs and GNCSs (mixtures of functionalized GNPs and both forms of cisplatin) were also probed. In performed analyses, applied concentrations of analytes were two times lower than those used previously.

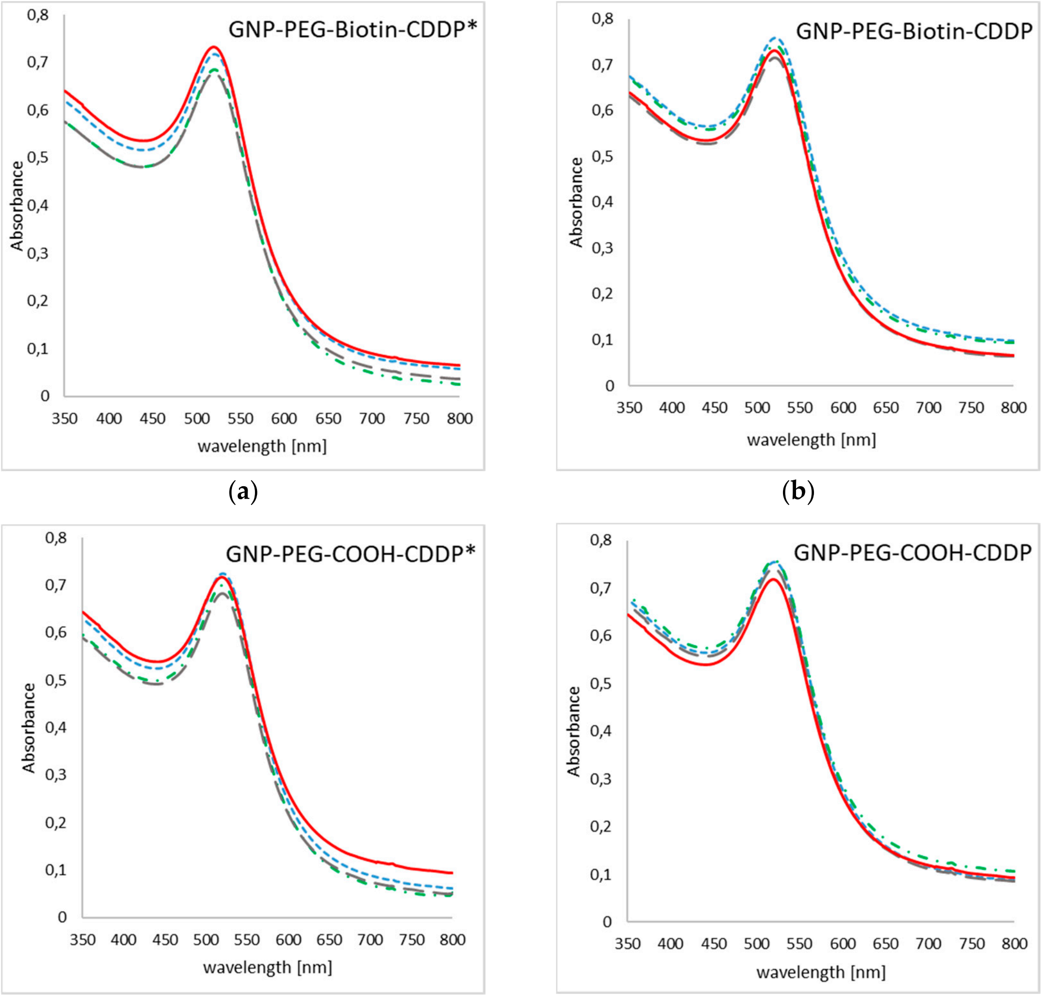

(**a**) (**b**)

(**c**) (**d**)

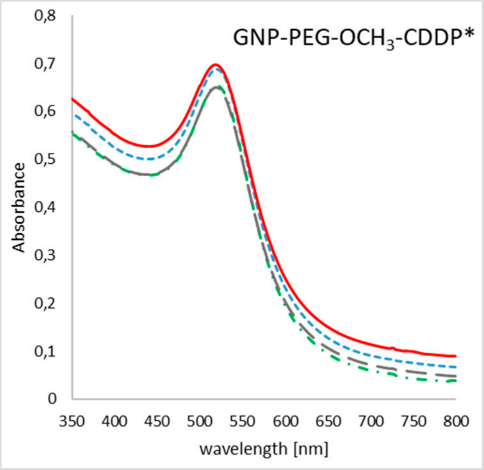

(e)

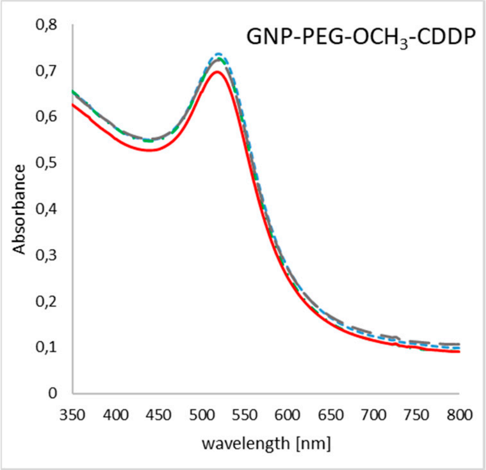

(f)

Figure S3. UV-Vis spectra of functionalized GNPs (control, the solid red line) and GNCSs samples (at optimized reagents ratio) in TB measured after various incubation times (4 h – the blue dotted line; 24 h – the green dash-dotted line; 48 h – the grey long dashed line): (**a**) GNP-PEG-biotin-CDDP*; (**b**) GNP-PEG-biotin-CDDP; (**c**) GNP-PEG-COOH-CDDP*; (**d**) GNP-PEG-COOH-CDDP; (**e**) GNP-PEG-OCH_3-CDDP*; (**f**) GNP-PEG-OCH_3-CDDP.

References

1. May, T.W.; Wiedmeyer, R.H. A table of polyatomic interferences in ICP-MS. *At. Spectrosc.* **1998**, *19*, 150–155.
2. Singh, P.; Pandit, S.; Mokkapati, V.R.S.S.; Garg, A.; Ravikumar, V.; Mijakovic, I. Gold nanoparticles in diagnostics and therapeutics for human cancer. *Int. J. Mol. Sci.* **2018**, *19*, 1979, doi:10.3390/ijms19071979.
3. Gong, Z.; Yang, R.; Sun, C.-Q.; Han, W.-N.; Jiang, X.-H.; Hu, S.-X.; Wang, Y. Simultaneous determination of P and S in human serum , blood plasma and whole blood by ICP-MS with collision/reaction cell technology. *Int. J. Mass Spectrom.* **2019**, *445*, 116193, doi:10.1016/j.ijms.2019.116193.
4. Persson, D.P.; Hansen, T.H.; Laursen, K.H.; Schjoerring, J.K.; Husted, S. Simultaneous iron, zinc, sulfur and phosphorus speciation analysis of barley grain tissues using SEC-ICP-MS and IP-ICP-MS. *Metallomics* **2009**, *1*, 418–426, doi:10.1039/b905688b.
5. Balcaen, L.; Bolea-Fernandez, E.; Resano, M.; Vanhaecke, F. Inductively coupled plasma - tandem mass spectrometry (ICP-MS/MS): a powerful and universal tool for the interference-free determination of (ultra)trace elements - a tutorial review. *Anal. Chim. Acta* **2015**, *894*, 7–19, doi:10.1016/j.aca.2015.08.053.
6. Machado, R.C.; Amaral, C.D.B.; Schiavo, D.; Nóbrega, J.A.; Nogueira, A.R.A. Complex samples and spectral interferences in ICP-MS: evaluation of tandem mass spectrometry for interference-free determination of cadmium, tin and platinum group elements. *Microchem. J.* **2017**, *130*, 271–275, doi:10.1016/j.microc.2016.09.011.
